# Atypical atrial flutter ablation: follow-up and predictors of arrhythmia recurrence

**DOI:** 10.1007/s00380-024-02417-2

**Published:** 2024-05-22

**Authors:** Peller Michał, Krzowski Bartosz, Rutkowski Kacper, Marchel Michał, Maciejewski Cezary, Mitrzak Karolina, Opolski Grzegorz, Grabowski Marcin, Balsam Paweł, Lodziński Piotr

**Affiliations:** Department of Cardiology, Medical University of Warsaw, Banacha 1a, 02-097 Warsaw, Poland

**Keywords:** Catheter ablation, Atypical atrial flutter, Atrial arrhythmia

## Abstract

Background: Ablation techniques have evolved greatly with advances in high-density 3D mapping systems over the last few years. Some patients develop atypical atrial flutter (AAFL) after pulmonary vein isolation (PVI). The data regarding follow-up after AAFL ablation as well as predictors of arrhythmia recurrence are lacking. This analysis aims to report procedure success rates and establish predictors of long-term success. Methods and Results: This retrospective cohort study included 45 patients (median age: 69 years; 40% female) who qualified for their first AAFL after PVI. The procedures were performed with the use of conventional ablation-index-guided ThermoCool Smarttouch SF and QDOT MICRO catheters. Freedom from arrhythmia recurrence was used as a primary end point. After 52 weeks of follow-up, 60% of patients suffered from arrhythmia recurrence, but over 70% of the studied cohort reported symptom improvement. In multivariate analysis, class I antiarrhythmics prescription (HR = 0.24 [95% CI 0.06–0.94], *p* = 0.04) was associated with the lack of arrhythmia recurrence during the follow-up, while cardioversion during procedure was associated with increased risk of arrhythmia recurrence (HR = 7.05 [95% CI 2.09–23.72], *p* = 0.002). Conclusions: Long-term success of AAFL ablation procedures is not satisfactory despite improvement in symptoms. Class I antiarrhythmics prescription at the discharge contributes to higher chances of sinus rhythm maintenance, whereas cardioversion during the procedure is related to increased risk of arrhythmia recurrence.

**Supplementary Information:**

The online version contains supplementary material available at 10.1007/s00380-024-02417-2.

## Introduction

Atrial fibrillation (AF) carries significant burden to patients, physicians, and healthcare systems globally. The risk for AF development during a lifetime has been established to be 1 in 3 individuals [1]. Pulmonary vein isolation (PVI) remains a cornerstone of AF treatment and is recommended at the early stages of the disease, as suggested by guidelines [1]. Atypical atrial flutter (AAFL) is defined by a macro-reentry mechanism, which is not dependent on the cavotricuspid isthmus. It is often related to structural heart disease, such as prior PVI, cardiac surgery, or prior surgical atriotomy [2]. However, AAFL can also occur as a result of idiopathic fibrotic atriopathy, without a history of previous procedures [3]. Patients with AAFL are often highly symptomatic and poorly respond to antiarrhythmic drugs [4], which leaves catheter ablation (CA) as one of the preferred treatment methods. Taking into consideration the rapidly growing population of patients undergoing PVI, more and more patients are suffering from AAFL. Considerable interest has been shown in novel mapping and ablation techniques, which could contribute to better prognosis. Mapping using high-density 3D mapping systems as well as ablation techniques evolved significantly over the last years enabling the treatment of complex atrial arrhythmias, including AAFL. Understanding of the arrythmia mechanism as well as the ablation approach has changed due to the introduction of electroanatomic high-density mapping systems. Using novel systems and catheters enables limiting entrainment, which may result in arrhythmia interruption, induction of AF, or cycle length change. Still, ablation of AAFL is often associated with prolonged procedure times and usually modest success rates. Commonly, the authors report results from small groups, with different follow-ups and procedure workflows. Peri-procedural success varies from 85 to 98%, but the long-term success rate ranges from 51 to 77% in patients after PVI [5–7]. Since AAFL are not highly common, even in big volume centers, the data regarding follow-up, as well as predictors of arrhythmia recurrence are scarce. The objective of this study was to describe the follow-up of patients with a history of PVI, who underwent AAFL ablation. We also aimed to establish predictors of arrhythmia recurrence.

## Materials and methods

### Study design

This is a retrospective cohort analysis conducted at a tertiary care center in Poland performing approximately 700 ablations per year. A total of 49 procedures of AAFL were performed between November 2019 and January 2023. Four procedures were excluded from this analysis because of second AAFL ablations. Patients were always qualified for the CA after at least a 3-month blanking period post-PVI. Every procedure was performed by experienced operators, who perform > 50 PVIs yearly. The inclusion criteria were: ≥ 18 years of age, history of PVI, and qualification for the first AAFL CA. Patients with an idiopathic AAFL or related to prior surgical procedures were excluded from this analysis. The study was conducted per the Declaration of Helsinki’s ethical principles. The protocol of the investigation was approved by the local Bioethics Committee (approval number: AKBE/127/2022). All patients signed informed consent to the processing of personal data.

### Procedural workflow

The symptoms were assessed with the EHRA scale: EHRA I—‘No symptoms’; EHRA II—‘Mild symptoms’; normal daily activity not affected; EHRA III—‘Severe symptoms’; normal daily activity affected; EHRA IV—‘Disabling symptoms’; normal daily activity discontinued. The majority of PVI procedure was preceded with trans-esophageal echocardiography (TEE) to rule out an intracardiac thrombus and assess the anatomy of the interatrial septum. In some low-risk patients, treated continuously with anticoagulants and with sinus rhythm on the day of the procedure TEE was revoked.

All catheters were inserted under local anesthesia through three vein punctures. During the procedure, unfractionated heparin was infused according to the activated coagulation time (target: > 335 s); the first bolus dose (120 IU/kg) was administered before trans-septal puncture. In all patients with left-sided arrhythmia double trans-septal puncture was performed with subsequent introduction of one unsteerable sheath for PentaRay NAV Eco catheter (Biosense Wenster Inc., CA, USA) and one steerable sheath for ThermoCool Smarttouch Surround Flow (SF) or QDot MICRO catheter (Biosense Wenster Inc., CA, USA). Intra-procedural pain was managed with an opioid (mostly continuous remifentanil infusion). Some patients required sedation with midazolam boluses introduced at an operator’s discretion. In most procedures, a three-dimensional reconstruction of the left atrium (LA) and pulmonary veins (PV) was created using rotational angiography if this has not been performed during previous PVI procedure. Bipolar voltage mapping was generated using a PentaRayTM catheter as well as a CARTO electroanatomic navigating system.

A total of 447 patients underwent AF ablation at the Medical University of Warsaw between November 2019 and January 2023, with 362 first-time sessions and 85 repeat sessions. From the group of patients who underwent first-time AF ablation, AAFL during initial PVI occurred in 7 patients (1.93%). AAFL was induced during maneuvering with catheters in 4 patients and in case of 3 patients AAFL was observed after AF cardioversion. No antazoline and no pacing maneuvers are performed in our center during first-time PVI procedure. On the other hand, of 38 patients with repeat ablation, PV reconnection was observed in 13 patients. After confirming or completing PV isolation, AAFL was induced by administration of antazoline in 4 patients; in 13 patients, AAFL was induced with pacing maneuvers. AAFL spontaneously occurred in the other 21 patients. Then, a total of 49 patients experienced AAFL in repeat sessions. Excluding 4 patients with second AAFL ablation, we examined a total of 45 patients (7 first-time and 38 repeat sessions) who underwent the first-time AAFL ablation. Moreover, it should be emphasized that as a referral center, some procedures were previously performed elsewhere.

For patients in sinus rhythm, stimulation maneuvers and isoprenaline infusion were applied for arrhythmia induction. For patients presenting in AAFL at the beginning of the procedure, the coronary sinus (CS) catheter activation was analyzed. In case of a distal to proximal activation, left atrial access was obtained and the left atrium (LA) was mapped first. In patients with a proximal to distal CS activation or whenever a right atrium (RA) AAFL was suspected, mapping of the RA was performed first. However, the trans-septal puncture was also performed even in right-sided ablations to ensure the durability of previously performed PVI. In patients with AF at the beginning of the procedure, antazoline was administered. Up to 400 mg during 10 min, infusion of antazoline has been administered. In the case of arrhythmia conversion from AF to AAFl, patients were included in this study. Local activation time mapping was performed with high-density point collection. Windows of interest were set to 90% of the total cycle length. CS signals were used for local activation point annotation. Both isochronal and propagation maps were generated at the discretion of the operator. Slow-conducting zones were defined by electrogram (EGM) of low-amplitude and isochronal crowding. Efforts were made to map the entire AAFL circuit. Voltage mapping was generally performed using < 0.05 mV as low voltage or scar and > 0.5 mV as healthy tissue. Moreover, entrainment was minimized and used in case of the inability to identify the AAFL circuit and critical isthmus with high-density mapping. Following beat, acceptance criteria were applied during automated point acquisition: (1) tachycardia cycle length stability ± 10 ms); (2) time stability of a reference EGM from the coronary sinus catheter (± 5 ms); (3) beat-to-beat EGM consistency; (4) EGMs acquired during the expiration phase; (5) EGMs acquired during stable position of the catheter (< 1 mm catheter movement during the acquisition window); and (6) tracking quality of the mapping catheter to ensure correct location of the mapping catheter. Manual re-annotations of doubtful EGMs were performed in a small subset of cases. In all patients, AP and RL diameters of LA were measured based on the electroanatomical map. Areas with voltage ≥ 0.3 mV during AF/AFL and > 0.5 mV during sinus rhythm have been qualified as low voltage. Patients were divided into two groups of low voltage (at least 25% of LA area with ow voltage) and regular voltage (the remaining part). Voltage areas were analyzed only if at least half of the expected chamber volume has been mapped.

AI-guided ablations with the use of Thermocool Smarttouch SF or QDot MICRO catheter were performed by adapting the CLOSE protocol. Power output was 35 W with a target AI of > 400 at the superior, posterior, and inferior wall of the left atrium and > 550 at the remaining sites. Other settings included: aimed CF of 10–30 g, irrigation rate of 15 mL/min, a maximum inter-lesion distance of 6 mm, and maximum temperature cut-off of 40 °C. Ablation of AAFL aimed to restore sinus rhythm and make a durable ablation line in the atrium, which would prevent arrhythmia recurrence. A two-sided conduction block was confirmed after the last application. Additionally, all patients had pulmonary veins checked and re-isolated in case of reconnection. Procedural success was confirmed when an entrance block to all pulmonary veins was proved. In each case, 15-min waiting period was ordered, when both stimulation maneuvers and isoprenaline infusion took place. If no arrhythmia was induced, the procedure was finished. Pacing maneuvers were made only in patients with history of AAFL. In case of arrhythmia induction, additional ablations were performed. If the arrhythmia was constantly inducible, it was left to the operator whether to continue procedure considering potential gain and risk of next ablations. However, in each case, cardioversion has been made before procedure completion. In 7/45 (15.6%) patients, AAFL occurred during the first PVI procedure. Based on the workflow used in our center, in case of registration of regular, sustained atrial arrhythmia, we perform the remap of the left atrium and ablate the arrhythmia substrate. In 13/45 (28.9%) patients, we performed re-isolation of pulmonary veins because of reconnections, which was always the first step of the procedure (before linear ablations).

Pericardial effusion and other intracardiac complications were excluded by transthoracic echocardiography performed twice: immediately after the procedure and in the morning of the following day, before being discharged from the hospital. Subsequent antiarrhythmic treatment was prescribed at the discretion of the operator.

### Study endpoints

The primary endpoint was freedom from any > 30 s, of atrial arrhythmia recorded on ECG after the procedure. The secondary aim of this study was to establish the predictors of arrhythmia recurrence. Additionally, patient satisfaction and symptoms after the CA were analyzed.

### Follow-up of the patients

All aforementioned information was extracted from the medical records. Clinical follow-up data were gathered during a routine post-discharge appointment at the outpatient clinic. Each patient had a 24-h Holter scheduled at 3 and 12 months after ablation. Then patients were contacted by telephone for additional information including any post-ablation symptoms of arrhythmia, documentation of potential recurrence, and precise time when it occurred. No blanking period was established.

### Statistical analysis

Distributions of continuous variables were assessed with the Shapiro–Wilk test. For variables with non-normal distribution, the results are presented as median and interquartile range, while variables with normal distribution are presented as mean ± standard deviation. Categorical variables are presented as percentages. Fisher exact test was used for comparing categorical variables, and Mann–Whitney *U* test was used for continuous variables. To assess the risk of atrial arrhythmia recurrences, univariate and multivariate stepwise Cox proportional hazards regressions were performed, assuming the p entry value as 0.2 and the p stay value as 0.1. To assess the influence of measured parameters on impedance level, Spearman correlations were calculated. Kaplan–Meier survival curves were plotted for an analysis of the atrial arrhythmia recurrences. A *p* value of < 0.05 was considered statistically significant. The statistical analysis was performed using Statistical Analysis Software (Cary, NC, USA), version 9.4.

## Results

### Patients’ characteristics

Data from 45 patients were included in this analysis after 233 PVI procedures. The median age was 69 (60–72) years, 40% were female, and the mean BMI was 28.03 ± 4.59. The majority of the population suffered from hypertension (78%), and every fifth patient suffered from heart failure. 18 (40%) patients had persistent AAFL. More than 90% of the population were treated with beta-blockers, over 17% of patients were on propafenone or flecainide, and only 3 patients were recommended to take amiodarone. No differences in the diameters of LA were observed. Detailed baseline population characteristics are presented in Table 1. There were no statistically significant differences in the univariate analysis between patients with and without arrhythmia recurrence. The median time of follow-up was 19 (5–40) weeks.

**Table 1:** General population characteristics

Variable		Overall population	Patients without arrhythmia recurrence during follow-up	Patients with arrhythmia recurrence during follow-up	*P* Value
Age [years]		69 [60–72]	69.5 [60–73]	68 [65–72]	0.94
Male		27 (60%)	13 (65.00%)	12 (48.00%)	0.37
Weigh [17]		82.32 ± 17.18	86.33 ± 16.75	79.44 ± 17.24	0.92
Height [cm]		170.86 ± 8.48	172.9 ± 8.99	169.4 ± 7.92	0.45
BMI [kg/m2]		28.03 ± 4.59	28.66 ± 3.97	27.57 ± 5.01	0.45
Hypertension		35 (77.78%)	17 (85.00%)	18 (72.00%)	0.47
Diabetes		8 (17.78%)	4 (20.00%)	4 (16.00%)	1
Dyslipidemia		27 (60%.00)	10 (50.00%)	17 (68.00%)	0.24
Chronic kidney disease		5 (11.11%)	3 (15.00%)	2 (8.00%)	0.64
Chronic coronary syndrome		12 (26.67%)	7 (35.00%)	5 (20.00%)	0.32
Heart failure		9 (20%)	5 (25.00%)	4 (16.00%)	0.48
Paroxysmal atrial fibrillation		20 (44.44%)	10 (50.00%)	10 (40.00%)	0.85
Persistent		25 (55.56%)	8 (40.00%)	17 (68.00%)	0.2
Antiarrhythmic treatment					
Class I antiarrhythmics		8 (17.78%)	5 (25.00%)	3 (12.00%)	0.44
Beta-blocker		42 (93.33%)	19 (95.00%)	23 (92.00%)	1
Anticoagulation					
Rivaroxaban		4 (8.16%)	2 (10.00%)	2 (8.00%)	0.87
Apixaban		23 (52.17%)	11 (55.00%)	12 (48.00%)	0.47
Dabigatran		15 (33.33%)	4 (20.00%)	11 (44.00%)	0.12
VKA		0	0	0	-
EHRA	1	2 (4.44%)	1 (5.56%)	1 (4.00%)	0.25
	2a	3 (6.67%)	1 (5.56%)	2 (8.00%)	
	2b	15 (33.33%)	4 (22.22%)	11 (44.00%)	
	3	20 (44.44%)	11 (61.11%)	9 (36.00%)	
	4	3 (6.67%)	1 (5.56%)	2 (8.00%)	

*AAFL* atypical atrial flutter; *BMI* body mass index; *EHRA* European Heart Rhythm Association; *VKA* vitamin K antagonist

#### Procedural’ characteristics

CA procedures performed on the analyzed cohort started mostly with an AAFL – 44%. The mean duration of the procedure was 183.33 ± 65.21 min, while the median fluoroscopy time reached 422.5 [299.0–629.0] s. On average, 74.88 ± 42.03 RF applications with a mean time of application of 29.63 ± 17.82 min were made during the CA. Acute peri-procedural success was achieved in 86.67% of patients. In 4 (8.89%) patients, post-procedural pericardial effusion was observed, without tamponade symptoms and need for intervention. No other complications were observed. There were no significant differences between patients who suffered from atrial arrhythmia recurrence during follow-up and those who did not. Detailed procedural data are presented in Table 2. Moreover, in 17 patients, 1 ablation line was made, while in 15 patients, 2 ablation lines were made. The remaining 13 patients had 3 or more ablation lines made. In the case of 3 mitral lines and 1 anterior line, the conduction block was not obtained. Further applications were abandoned due to fear of peri-procedural complications. In 14 (31.11%) patients, re-isolation of the pulmonary veins was performed.

**Table 2:** Procedural characteristics

Variable	Overall population	Patients without arrhythmia recurrence during follow-up	Patients with arrhythmia recurrence during follow-up	*P* Value
Procedure time [min]	183.3 ± 65.2	184.4 ± 72.19	182.6 ± 61.5	0.93
Fluoroscopy time [sec]	509.4 ± 309.5	516.1 ± 273.1	505.0 ± 336.7	0.83
Ablation time [min]	14.84 ± 9.25	13.80 ± 8.08	15.56 ± 10.07	0.52
Number of RF applications	74.88 ± 42.03	77.67 ± 53.21	72.96 ± 39.4	0.74
Sinus rhythm at the beginning of the procedure	16 (35.56%)	7 (35.00%)	9 (36.00%)	1
Right–left atrial diameter [mm]	69.44 ± 10.39	72.41 ± 10.83	66.88 ± 9.50	0.09
Anterior–posterior atrial diameter [mm]	44.84 ± 10.03	46.06 ± 9.35	43.83 ± 10.67	0.49
Number of patients with > 25% of low voltage area in the left atrium	26 (63.41%)	11 (26.83%)	15 (36.59%)	0.53
Ablation lines				
Cavo-tricuspid isthmus	11 (24.44%)	6 (30.00%)	5 (20.00%)	0.50
Anterior line	30 (66.67%)	14 (70.00%)	16 (64.00%)	0.76
Lateral line	2 (4.44%)	2 (10.00%)	0	0.19
Roofline	25 (55.56%)	11 (55.00%)	14 (56.00%)	1
Mitral isthmus	9 (20%)	2 (10.00%)	7 (28.00%)	0.26
Posterior line	15 (33.33%)	8 (40.00%)	7 (28.00%)	1
Septal line	5 (11.11%)	3 (15.00%)	2 (8.00%)	0.64
Box	2 (4.44%)	1 (5.00%)	1 (4.00%)	1
Cardioversion during procedure	6 (13.33%)	1 (5.00%)	5 (20.00%)	0.20
Conversion to sinus rhythm during procedure	39 (86.67%)	19 (95.00%)	20 (80.00%)	
Peri-procedural complications				
Post-procedural pericardial effusion	4 (8.89%)	1 (5.00%)	3 (12.00%)	0.62
Vascular complications	0	0	0	-
Cerebrovascular event	0	0	0	-
Follow-up				
Atrial arrhythmia recurrence before discharge	4 (8.89%)	1 (5.00%)	3 (12.00%)	0.62

*AAFL* atypical atrial flutter

### Clinical outcomes and atrial arrhythmia recurrence predictors

**Fig. 1:**
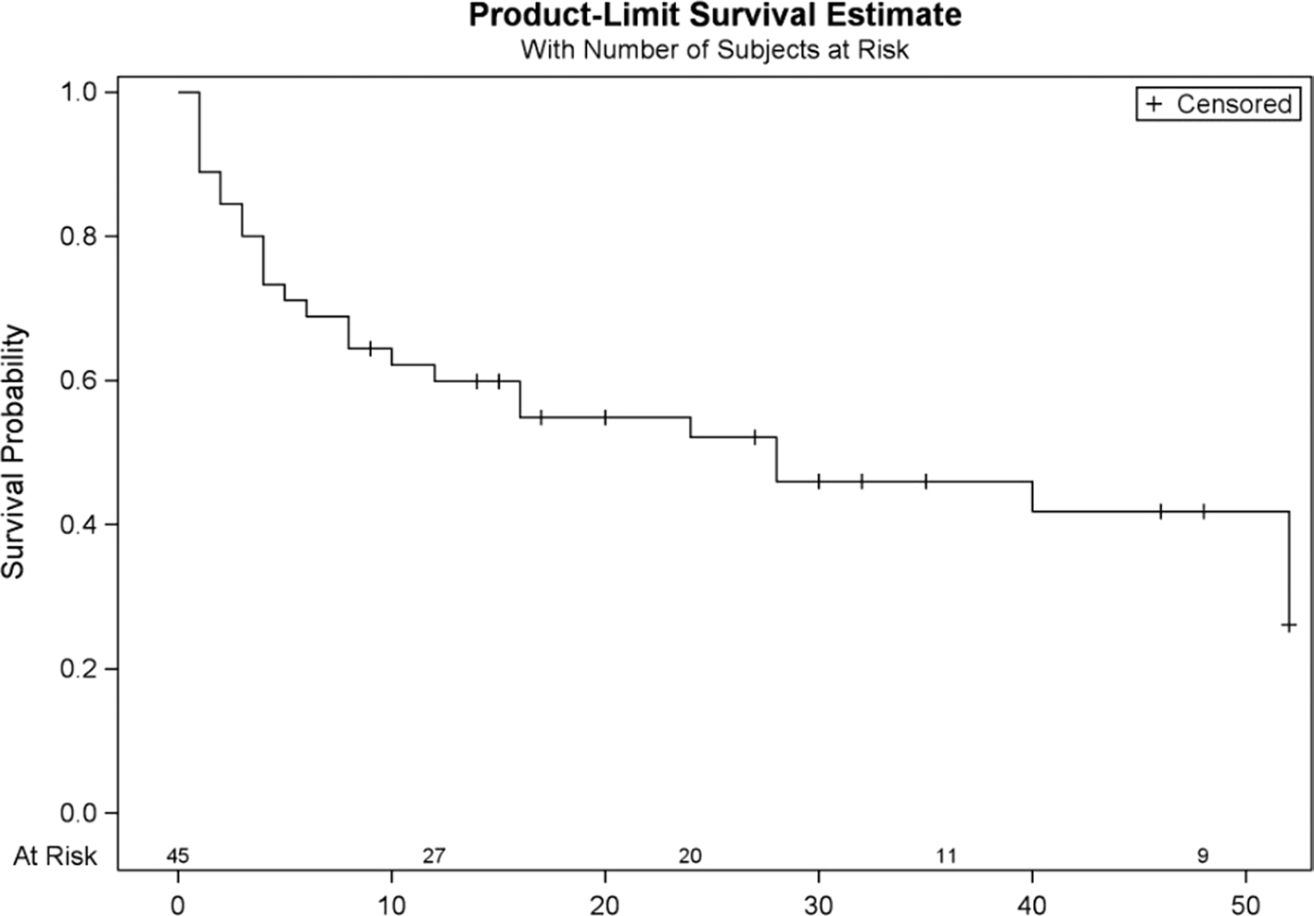
Arrhythmia recurrence during follow-up

**Fig. 2:**
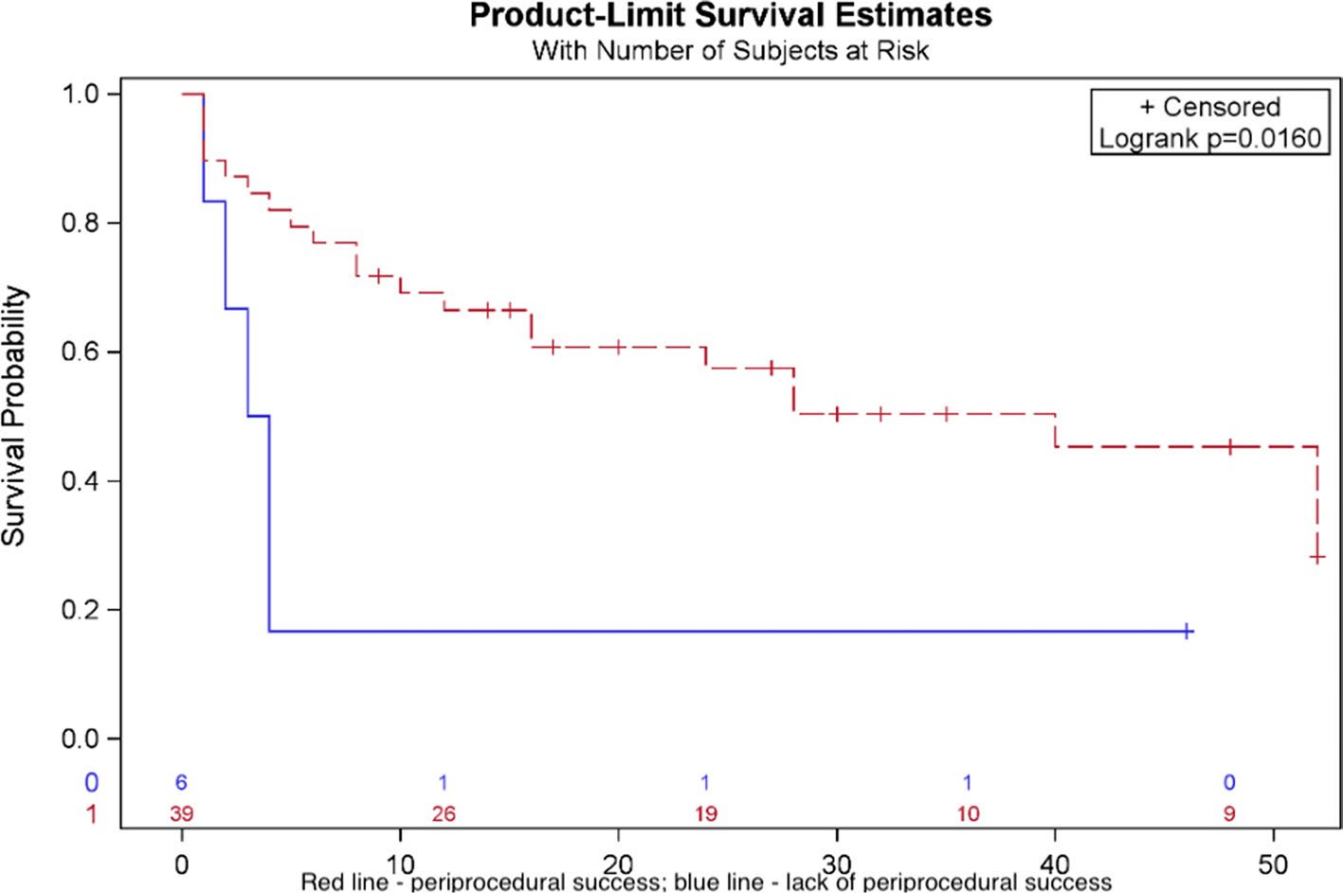
Probability of atrial arrhythmia recurrence by the acute peri-procedural success

**Fig. 3:**
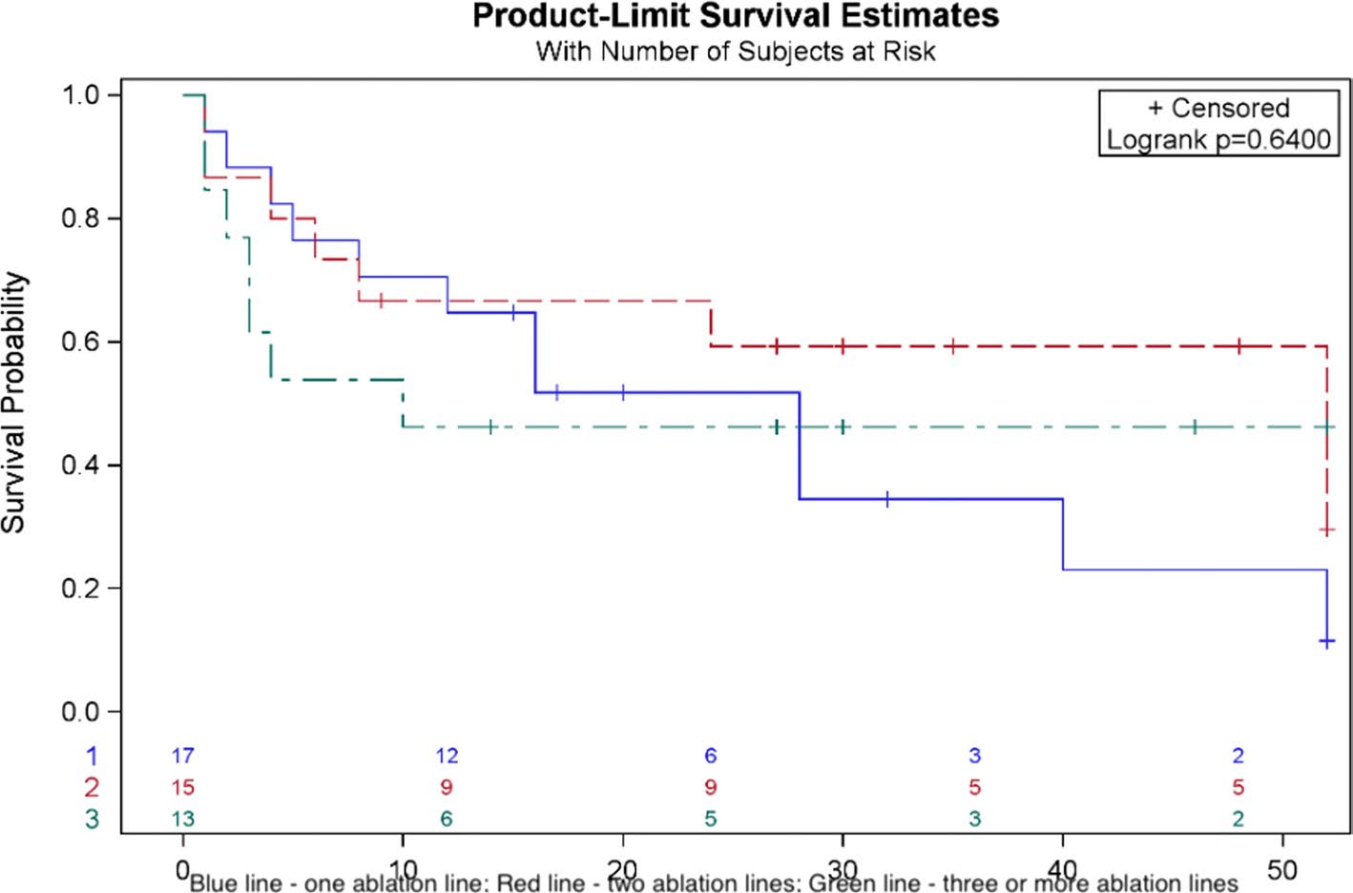
Probability of atrial arrhythmia recurrence by the number of ablation lines

**Fig. 4:**
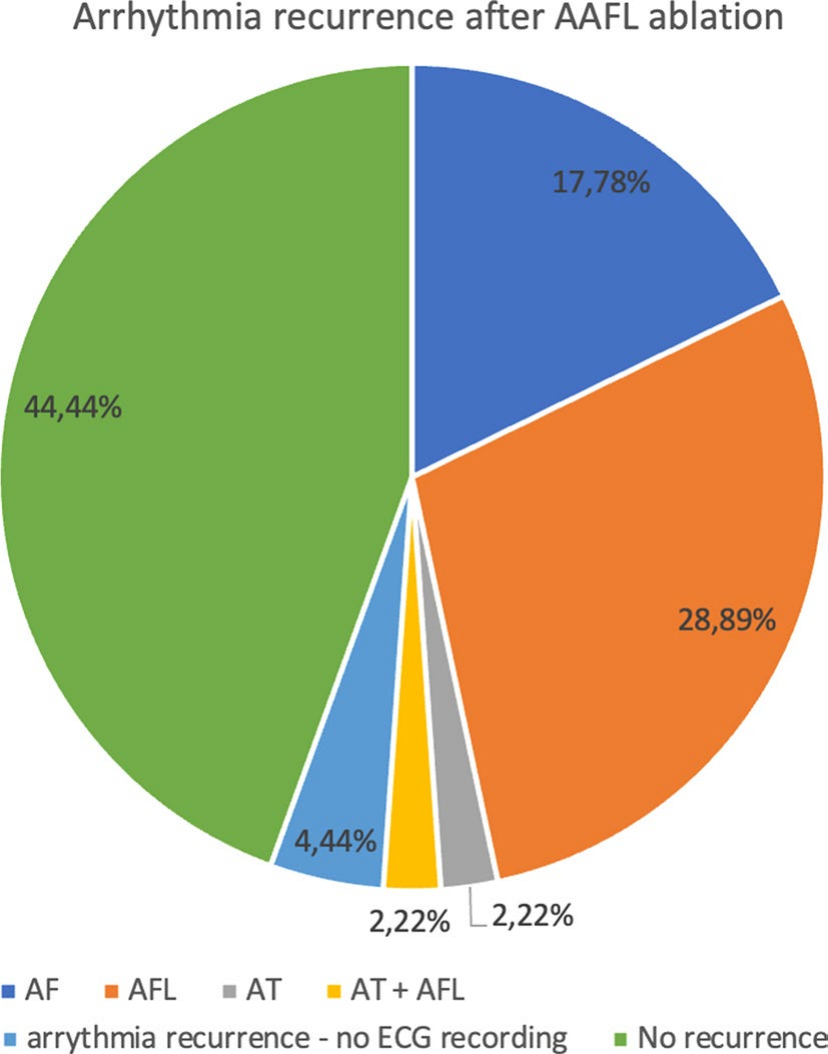
Atrial arrhythmia recurrence after AAFL ablation

No long-term complications were observed. Atrial arrhythmia recurrence was found in 25 (55.6%) patients, in 4 of them (8.89%) even before discharge from the hospital. More than half of the studied cohort did not suffer from any form of atrial arrhythmia after 6 months (Fig. 1). Univariate analysis showed that patients with acute peri-procedural success and sinus rhythm restoration during applications have a higher likelihood of remaining free from atrial arrhythmia during the follow-up (Fig. 2), which has not been confirmed in the multivariable analysis. Risk factors of arrhythmia recurrence in univariate analysis have been summarized in supplementary Table S1. Moreover, the number of lines performed during CA did not affect long-term success (Fig. 3). Also, the number of AAFL observed during the procedure did not impact the arrhythmia recurrence rate during follow-up (p = 0.42) (Figure S1), as well as extensive areas of low voltage in the left atrium showed no correlation with arrhythmia recurrence (*p* = 0.43) (Figure S2). In multivariate analysis, only class I antiarrhythmics prescription HR = 0.24 [95% CI 0.06–0.94], *p* = 0.04) was associated with the lack of arrhythmia recurrence during the follow-up, while cardioversion during the procedure was associated with increased risk of arrhythmia recurrence (HR = 7.05 [95% CI 2.09–23.72], *p* = 0.002). The stepwise multivariate regression analysis model included three variables: cardioversion during procedure, class I antiarrhythmic prescription, and weight (which was not statistically significant). Every third patient had a recurrence of AF, and every fourth had a recurrence of AFL as a dominant atrial arrhythmia type (Fig. 4).

## Discussion

Major findings of our analysis are as follows: (1) the rate of atrial arrhythmia recurrence during the follow-up is relatively high; (2) patients with acute peri-procedural success tend to have better prognosis regarding arrhythmia recurrence; (3) class I antiarrhythmics prescription was associated with longer sinus rhythm maintenance; (4) CA for AAFL using a contemporary strategy mainly based on high-density mapping is associated with low risk of serious peri-procedural complications.

CA is constantly becoming increasingly popular since it has proven effective and safe [1, 8]. A variety of different arrhythmias can be treated with this method. Currently, the most significant technological progress can be observed in AF ablation, which results in a growing interest in the procedure. Unfortunately, some AF procedures can be complicated with AAFL occurrence during the follow-up. According to the available literature, recurrent AAFL has been reported to occur in 8–53% of patients after ablation and/or surgical procedures and varies between center, ablation method, and timing of the procedure. The substrate of the arrhythmia differs between patients after cardiosurgical interventions, where the atrium is usually relatively healthy, and post-PVI patients, where due to underlying disease development, left atrium is often significantly remodeled [9, 10]. The clinical presentation of this rhythm disturbance depends on several variables, but AV conduction is one of the most important one. One-to-one conduction might result in high ventricular rates with moderate clinical tolerance, often requiring rapid medical intervention.

Moreover, similarly to AF, during AAFL, ineffective atrial contraction appears to add to the hemodynamical distraction. As a result, patients may develop hypotension, heart failure exacerbation, syncope, decrease in physical activity tolerance, or occasionally, do not report any complaints [11]. Resistance of AAFL to pharmacological treatment prompts patients and physicians for another CA procedure with the aim of restoration and maintenance of sinus rhythm. The data on AAFL treatment are scarce. The objective of this analysis was to report follow-up of the patients, establish predictors of CA success, and study the procedure’s safety.

AAFL has been documented to occur in roughly 8% of patients with a history of prior ablation of CA. In a study by Akhtar et al., the authors investigated patients who had isolated pulmonary veins and developed AAFL or recurrent AF. Successful ablation of this arrhythmia has been achieved in 73.3% of patients. In those groups, the recurrent connection of pulmonary veins was observed in 76.4% of patients, which is way higher than reported in this manuscript, 31.11%, even though acute procedure success was higher in our analysis. AAFl has reoccurred in 12.7% of patients. It has been found that left atrial diameter and volume were greater in patients with the recurrence of AAFl than in patients who developed AF after prior ablation of the atrium [10]. We did not analyze this variable, but interestingly, contrary to our findings, male sex or antiarrhythmics prescription was not associated with arrhythmia-free survival in this analysis. It has also been stated that AAFL is more likely to occur after RF ablation rather than cryoablation. Unfortunately, we cannot refer to it since the vast majority of PVIs are done with RF in our center. In the different analyses performed by Chae et al., re-entrant septal AT was associated with arrhythmia recurrence, while PV isolation during the atrial tachycardia ablation was associated with a favorable outcome [7]. The heterogeneity of arrhythmia-free survival predictors could indicate that the analyzed cohorts are different and difficult to compare between studies. The long-term success rate of AAFL CA ranges from 60 to 75% [5–7, 12, 13], which is somewhat higher than in this analysis, but again the diversity of the treated population as well as methods of arrhythmia recurrence detection can impact the results significantly.

The data regarding procedure safety are lacking but remain important in assessing the risk-and-benefit ratio when scheduling patients for the next CA. Cardiac perforation is one of the most dangerous and possibly lethal complications of CA. The risk of this event increases with an increase in application count, and in AAFL CA, lots of applications are implemented. Early reports have documented the incidence rate as high as 6% after PVI. However, newer publications and a Germany-wide analysis have reported improved safety and a reduction in the prevalence of perforation to 0.61–0.9%. Female sex, obesity, and the lack of intracardiac echocardiography have been reported to be associated with a higher risk of cardiac peroration. Additionally, low-volume centers and radiofrequency ablation have been linked with higher rates of pericardial effusion [14, 15]. Fortunately, in our cohort, no tamponade was observed; however, 8.89% of patients had post-procedural pericardial effusion, none of which required any treatment. Anticoagulation has not been interrupted, which might have also played a role in the lack of another CA-related complication in the studied group—ischemic cerebral events. Ischemic stroke after AF CA is a complication with an incidence rate of 0.49%, but silent ischemia seems to be far more prevalent. Presence of late recurrence, bigger left atrium size, older age, non-paroxysmal AF, and decreased flow velocity in the left atrial appendage have been identified as risk factors of ischemic stroke in patients who have undergone the ablation of AF [16]. Patients scheduled for AAFL seem to have somehow more comorbidities when compared to typical patients scheduled for PVI. Therefore, one could expect that AAFL CA carries a bigger higher risk, which has not been observed in this analysis. However, the studied group is relatively small and possibly underpowered to detect some complications; therefore, pooling this data with information from other centers would broaden the perspective. The primary endpoint of this study was set as freedom from any atrial arrhythmia lasting more than 30 s on an electrocardiogram. Not using intracardiac monitoring could be regarded as a study limitation. Moreover, lack of data from ECHO should be regarded as a limitation. This issue has been addressed by showing analysis made based on the diameters obtained from electroanatomical maps, which might better reflect on the current size of the left atrium. The need for cardioversion may indicate the presence of multiple AAFL types or an AAFL subtype that is resistant to ablation. However, since the decision to perform cardioversion depends on the operator's discretion, bias might have occurred. On the other hand, each application might cause complications, especially in the fibrotic atrium. Therefore, cardioversions were performed when operator decided that the risk of further applications is bigger than potential benefit, which is another limitation of this analysis.

## Conclusions

CA for AAFL is associated with a moderate arrhythmia recurrence rate. Patients who had successfully ablated the substrate of arrhythmia, who are prescribed Class I AAD, are more likely to maintain sinus rhythm. Moreover, AAFL CA can be carried out safely without too much risk for an individual patient. Those results could contribute as a part of a larger, multicenter analysis in future or be a part of a meta-analysis of multiple studies reporting mixed results.

## Supplementary Information

Below is the link to the electronic supplementary material.

Supplementary file1 (JPG 385 KB)

Supplementary file2 (JPG 304 KB)

Supplementary file3 (DOCX 18 KB)

## Data Availability

The data that support the findings of this study are available on request from the corresponding author. The data are not publicly available due to reasons of sensitivity.
