# Supplementary figures and images for: Atypical atrial flutter ablation: follow-up and predictors of arrhythmia recurrence
Source: Heart Vessels. 2024 May 22;39(11):949–57. doi: 10.1007/s00380-024-02417-2 (PMC11489262; doi:10.1007/s00380-024-02417-2)

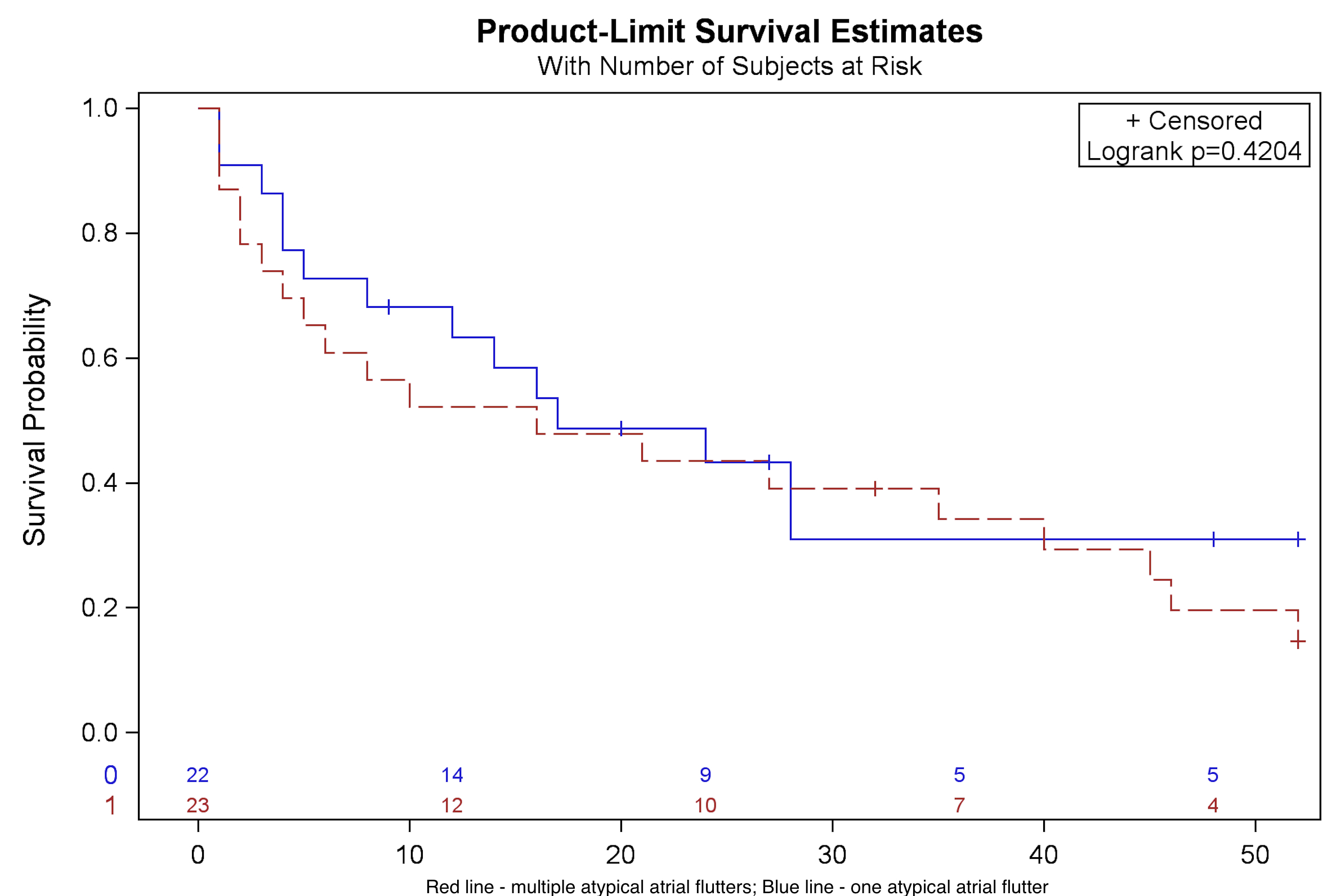

Supplement: Supplementary file 1 — Supplementary file1 (JPG 385 KB) [file 380_2024_2417_MOESM1_ESM.jpg]

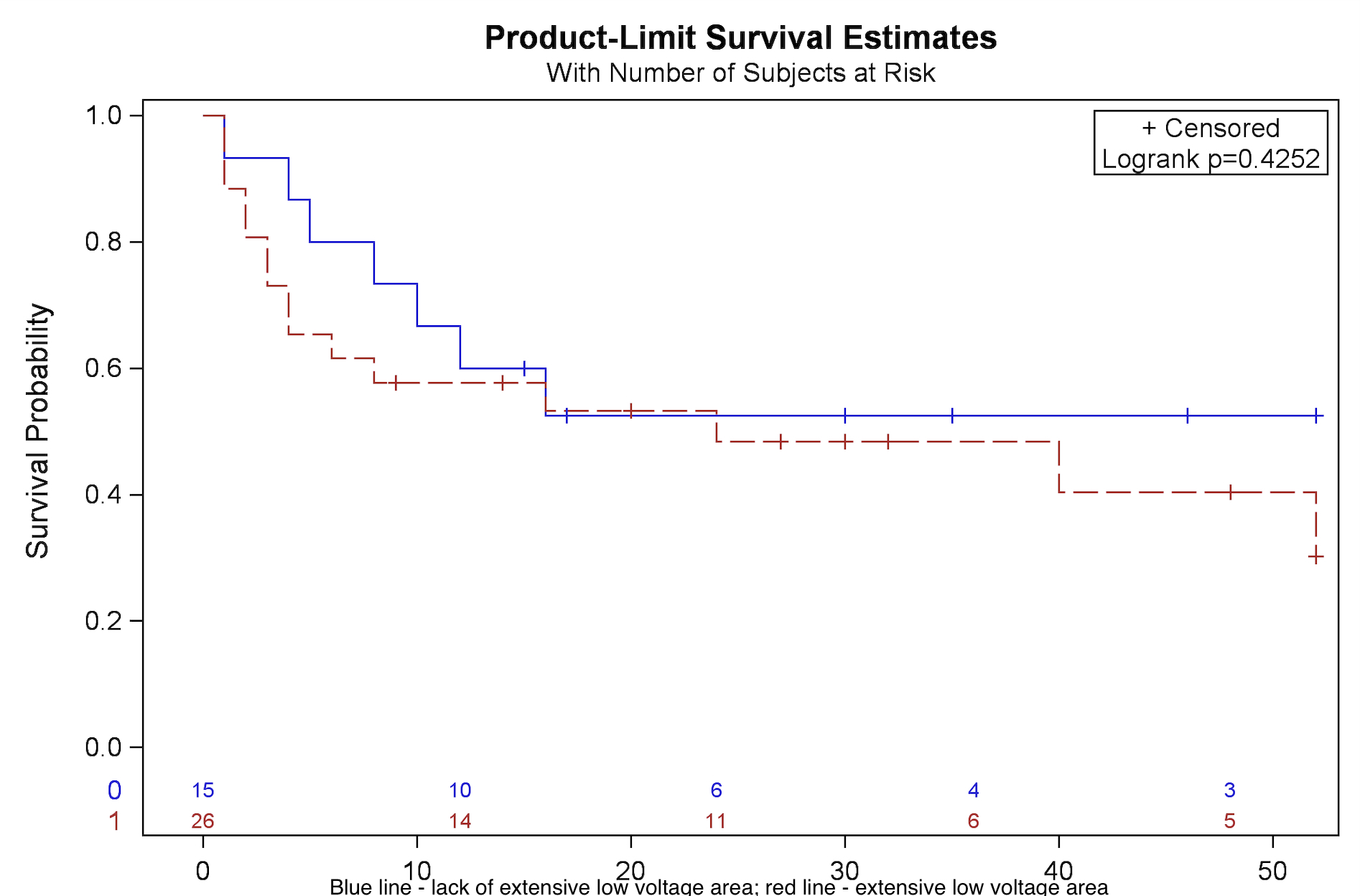

Supplement: Supplementary file 2 — Supplementary file2 (JPG 304 KB) [file 380_2024_2417_MOESM2_ESM.jpg]
