# Supplementary material for: Atypical atrial flutter ablation: follow-up and predictors of arrhythmia recurrence
Source: Heart Vessels. 2024 May 22;39(11):949–57. doi: 10.1007/s00380-024-02417-2 (PMC11489262; doi:10.1007/s00380-024-02417-2)
Supplement: Supplementary file 3 — Supplementary file3 (DOCX 18 KB) [file 380_2024_2417_MOESM3_ESM.docx]

Table S1. Risk factors of arrhythmia recurrence in univariate and multivariate analysis.

| Risk factor | Univariate analysis | | Multivariate analysis | |
| --- | --- | --- | --- | --- |
|  | HR (95% CI) | p-value | HR (95% CI) | p-value |
| Baseline characteristics | | | | |
| Age | 1.10 (0.82-1.63) | 0.53 |  |  |
| Sex male | 0.57 (0.27-1.23) | 0.15 |  |  |
| Weight [per 10kg] | 0.98 (0.96-1.01) | 0.14 | 0.97 (0.94-1.00) | 0.07 |
| BMI | 0.95 (0.87-1.04) | 0.28 |  |  |
| Diabetes | 0.6 (0.21-1.75) | 0.35 |  |  |
| Kidney disease | 0.7 (0.16-3.0) | 0.63 |  |  |
| Coronary artery disease | 0.46 (0.17-1.22) | 0.12 |  |  |
| Myocardial infarction history | 0.28 (0.04-2.13) | 0.22 |  |  |
| Heart failure | 0.59 (0.20-1.71) | 0.33 |  |  |
| Amiodarone | 0.66 (0.09-4.88) | 0.68 |  |  |
| Class I antiarrhythmics | 0.63 (0.22-1.82) | 0.39 | 0.24 (0.06-0.94) | 0.04 |
| Beta-adrenolytic | 1.05 (0.25-4.44) | 0.95 |  |  |
| Procedural parameters | | |  |  |
| Procedure time per 10 min | 0.98 (0.923-1.05) | 0.62 |  |  |
| Number of RF applications [per 10 points] | 0.99 (0.90-1.07) | 0.74 |  |  |
| Atrial arrhythmia recurrence before discharge | 2.57 (0.75-8.61) | 0.14 |  |  |
| Re-PVI | 0.61 (0.25-1.53) | 0.29 |  |  |
| First PVI | 1.19 (0.47-3.0) | 0.71 |  |  |
| Anterior septal line | 0.91 (0.38-2.16) | 0.83 |  |  |
| Box | 0.69 (0.09-5.11) | 0.71 |  |  |
| Mitral line | 0.97 (0.44-2.13) | 0.94 |  |  |
| Cavo-tricuspid isthmus | 0.64 (0.24-1.70) | 0.37 |  |  |
| Anterior line | 0.91 (0.40-2.06) | 0.82 |  |  |
| Roof line | 1.17 (0.52-2.39) | 0.78 |  |  |
| Posterior line | 0.71 (0.29-1.71) | 0.44 |  |  |
| Septal line | 0.72 (0.17-3.06) | 0.65 |  |  |
| Cardioversion during procedure | 3.15 (1.15-8.86) | **0.03** | 7.05 (2.09-23.72) | 0.002 |

Abbreviations: HR – hazard ratio; BMI – body mass index; RF – radiofrequency
